# Role of mitochondrial reactive oxygen species in age-related inflammatory activation of endothelium

**DOI:** 10.18632/aging.100685

**Published:** 2014-08-13

**Authors:** Roman A. Zinovkin, Valeria P. Romaschenko, Ivan I. Galkin, Vlada V. Zakharova, Olga Yu. Pletjushkina, Boris V. Chernyak, Ekaterina N. Popova

**Affiliations:** ^1^Belozersky Institute of Physico-Chemical Biology, Lomonosov Moscow State University, Vorobyevy Gory 1, Moscow 119991, Russia; ^2^Faculty of Bioengineering and Bioinformatics, Lomonosov Moscow State University, Vorobyevy Gory 1, Moscow 119991, Russia; ^3^Institute of Mitoengineering, Lomonosov Moscow State University, Vorobyevy Gory 1, Moscow 119991, Russia

**Keywords:** mitochondrial reactive oxygen species, endothelium, TNF, NF-kB, cell adhesion molecules

## Abstract

Vascular aging is accompanied by increases in circulatory proinflammatory cytokines leading to inflammatory endothelial response implicated in early atherogenesis. To study the possible role of mitochondria-derived reactive oxygen species (ROS) in this phenomenon, we applied the effective mitochondria-targeted antioxidant SkQ1, the conjugate of plastoquinone with dodecyltriphenylphosphonium. Eight months treatment of (CBAxC57BL/6) F1 mice with SkQ1 did not prevent age-related elevation of the major proinflammatory cytokines TNF and IL-6 in serum, but completely abrogated the increase in adhesion molecule ICAM1 expression in aortas of 24-month-old animals. In endothelial cell culture, SkQ1 also attenuated TNF-induced increase in ICAM1, VCAM, and E-selectin expression and secretion of IL-6 and IL-8, and prevented neutrophil adhesion to the endothelial monolayer. Using specific inhibitors to transcription factor NF-κB and stress-kinases p38 and JNK, we demonstrated that TNF-induced ICAM1 expression depends mainly on NF-κB activity and, to a lesser extent, on p38. SkQ1 had no effect on p38 phosphorylation (activation) but significantly reduced NF-κB activation by inhibiting phosphorylation and proteolytic cleavage of the inhibitory subunit IκBα. The data indicate an important role of mitochondrial reactive oxygen species in regulation of the NF-κB pathway and corresponding age-related inflammatory activation of endothelium.

## INTRODUCTION

Cardiovascular diseases (CVDs) have a great impact in morbidity and mortality all over the world. One of the major risk factors for development of CVDs is aging. In recent years a vast amount of information has been obtained pointing to a crucial role of endothelium in the development of age-related CVDs. A healthy endothelium fulfils numerous functions in vascular biology including inflammatory responses, as well as vascular tone and permeability. Endothelial dysfunction is typical for many pathological conditions including atherosclerosis, type I and II diabetes, inflammatory processes, and aging [1]. Aging is associated with increased oxidative stress and a proinflammatory endothelial cell phenotype. Excessive or prolonged endothelium activation due to the action of the proinflammatory cytokines underlies endothelium dysfunction [2]. TNF, IL-6, and other proinflammatory cytokines stimulate expression of cell adhesion molecules (CAMs) and promote leukocyte adhesion and transmigration. Aging is accompanied by increase in both circulatory TNF and expression of endothelium cell adhesion molecules [3, 4]. TNF-induced signaling pathways involved in inflammatory reactions are studied extensively [5]. Generally, TNF-activated signaling cascades lead to the activation of caspases and two transcription factors, AP-1 and NF-κB [6]. Expression of CAMs is regulated mostly via NF-κB and, to a lesser extent, via MAPK kinases p38 and JNK activating AP-1 [7, 8]. The NF-κB pathway plays a crucial role in age-related endothelial dysfunction [9].

In various cell types, mitochondria modulate the inflammatory response [10-12]. Though endothelial mitochondria do not play a significant role in ATP production, information is emerging indicating that they are important agents contributing to endothelial physiology and pathophysiology [12]. The mito-chondrial respiratory chain is a well-known source of ROS under various physiological conditions, and TNF can stimulate mitochondrial ROS (mtROS) production [13, 14]. mtROS are involved in a plethora of signaling pathways including TNF-induced signaling [15-18]. It is generally accepted that the NF-κB signaling pathway includes several redox-sensitive components (PTEN, SHIP-1, PP2A, and IKKα and β); however, controversy exists regarding the role of ROS in regulation of NF-κB signaling (reviewed in [19]). Moreover, the role of mtROS in endothelial activation, expression of CAMs, and the NF-κB signaling cascade is not fully understood.

Mitochondria-targeted antioxidants are powerful tools for investigating the role of mtROS in many processes *in vitro* and *in vivo* [20-25]. In the current study, we used SkQ1 antioxidant, based on the plastoquinone moiety linked to dodecyltriphenylphosphonium cation that targets SkQ1 to mitochondria [26]. SkQ1 and its analogs are efficient in the prevention of some age-associated pathologies, and they have therapeutic effects in animal models of diseases associated with inflammatory response (heart, brain, and kidney ischemic injury [27, 28], pyelonephritis [29], eye diseases [30, 31], sarcopenia [32], and dermal wound healing [33]). SkQ1 delays the development of various markers of aging and prolongs the lifespan of various animals [20, 22, 34, 35]. The antiinflammatory and vasoprotective action of SkQ1 could underlie some of these effects.

Using mitochondria-targeted antioxidants, we show that mtROS are critical for the increase in CAM expression both *in vivo* in aortas of old mice and *in vitro* in endothelial cells treated with TNF acting through the NF-κB pathway.

## RESULTS

### Mitochondria-targeted antioxidant SkQ1 inhibits expression of adhesion molecules ICAM1 in the aortas of old mice

**Figure 1:**
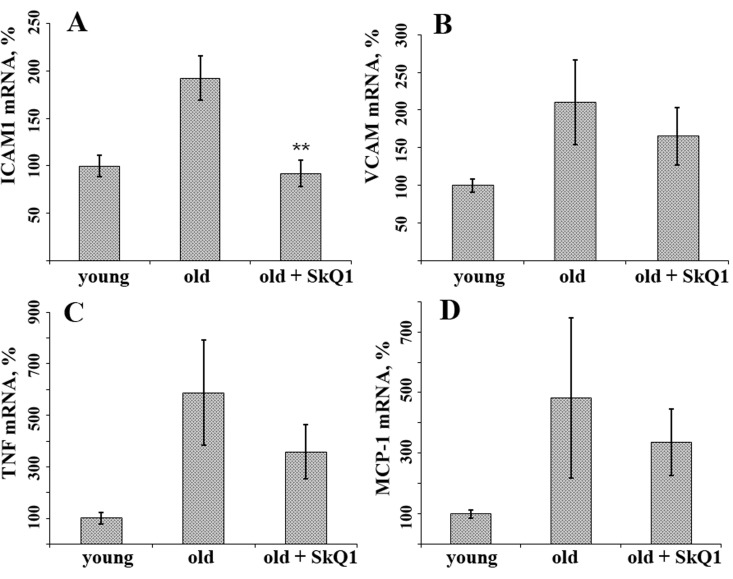
SkQ1 suppresses age-related increase in mRNA expression of some inflammatory markers in aortas of old mice (**A**) ICAM1; (**B**) VCAM; (**C**) TNF; (**D**) MCP-1. The animals were treated as indicated on Fig. 1. Data are represented as mean +/− SEM. n = 10. ** p < 0.001.

In aortal tissue of old (24 month) CBAxC57bl/6 mice, mRNA expression of inflammatory markers such as adhesion molecules ICAM1 and VCAM and cytokines TNF and MCP1 were higher than in young (8-month-old) animals (Fig. 1). Long-term consumption of the mitochondria-targeted antioxidant SkQ1 (100 nmol/kg body weight per day, 8 months) decreased mRNA expression of ICAM1 to the level of young animals (Fig. 1). SkQ1 also slightly decreased expression of other markers of inflammation, though these effects were not statistically significant. In old mice the levels of the inflammatory cytokines TNF and IL-6 in the blood plasma were higher than in young animals, in agreement with previously published data [36-38]. SkQ1 treatment did not significantly influence the level of these cytokines in old mice (Fig. 1). Thus, SkQ1 did not suppress the generation of TNF and IL-6, but it inhibited the activation effect of these cytokines in the aortic tissue. These data suggest that mtROS are involved in inflammatory response of endothelium in old mice.

**Figure 2:**
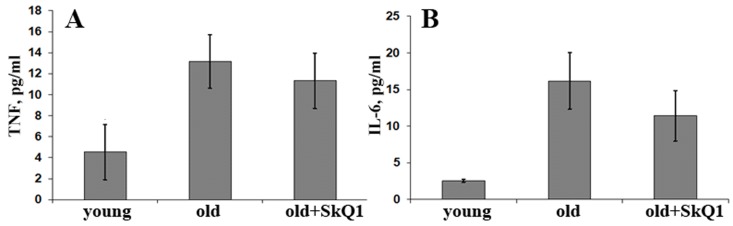
Long-term SkQ1 treatment (250 nmol/kg per day, 8 months) does not affect circulatory levels of proinflammatory cytokines in old (24 months) versus young (6 months) mice (**A**) TNF; (**B**) IL-6. Data are represented as mean +/− SEM. n = 10.

### SkQ1 inhibits TNF-induced activation of endothelium

To study the role of mtROS in the inflammatory response of endothelium, we investigated the effect of SkQ1 on activation of endothelial cells in culture stimulated with TNF. TNF is widely used to study inflammatory response *in vitro* in both primary cell cultures and in immortalized cell lines. In our work, we used the primary endothelial cell culture HUVEC and the immortalized EA.hy926 cell line established as a suitable model in many studies [39, 40].

TNF-induced endothelial activation was assessed using the following criteria: (i) increase in mRNA expression level of adhesion molecules ICAM, VCAM, and E-selectin; (ii) increase in ICAM1 expression on the surface of cells; (iii) increase in IL-6 and IL-8 secretion; and (iv) increase in adhesion of human promyelocytic leukemia cells (HL-60) to the endothelial monolayer.

### SkQ1 suppresses TNF-induced mRNA expression of adhesion molecules

**Figure 3:**
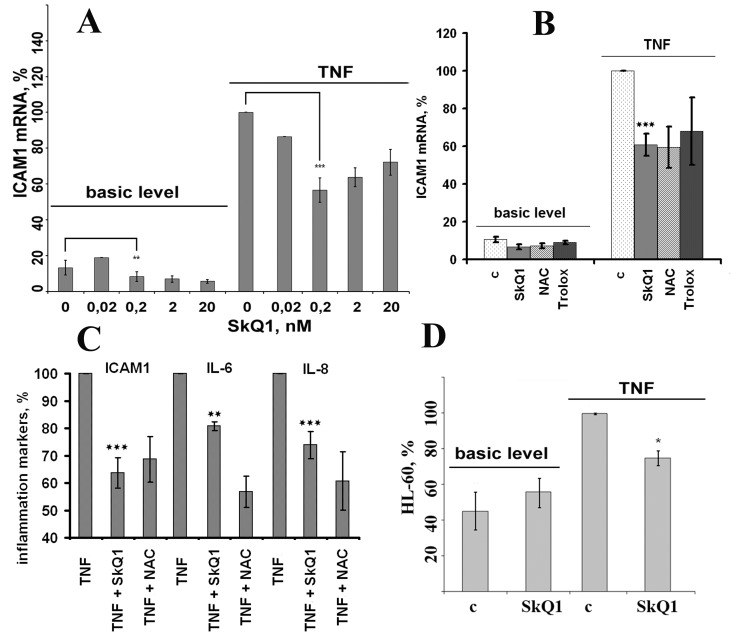
Antioxidants (0.2 nM SkQ1, 1 mM NAC, 200 μM Trolox) prevent TNF-induced activation of EA.hy926 endothelial cells (**A**, **B**) Expression of ICAM1 mRNA in TNF-induced (4 h, 50 pg/ml) endothelial cells. (**C**) Cell-surface ICAM1 expression and IL-6 and IL-8 cytokine secretion in TNF-induced (8 h, 5 ng/ml) endothelial cells (n = 3). (**D**) Neutrophil adhesion to endothelium monolayer stimulated with TNF (8 h, 5 ng/ml). c, control. Data are represented as mean +/− SEM; n = 3 except for 0.2 nM SkQ1 data on Figs. 3A-3C, where n ≥ 15. * p ≤ 0.05, ** p < 0.001,*** p < 0.0001.

**Figure 4:**
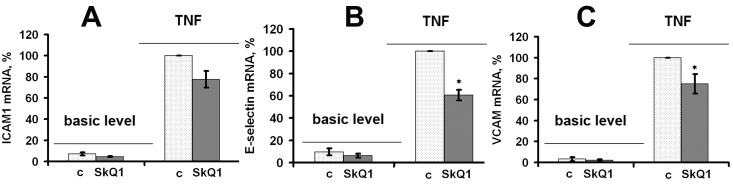
SkQ1 suppresses TNF-induced (4 h, 50 pg/ml) mRNA expression of cell adhesion molecules in HUVEC; c, control (**A**) ICAM1; (**B**) E-selectin; (**C**) VCAM. Data are represented as mean +/− SEM. n = 3. * p ≤ 0.05.

TNF (50 pg/ml) drastically increased mRNA expression of ICAM1 in both HUVEC and EA.hy926 endothelial cells (Fig. 3A, 4A). TNF also significantly increased expression of E-selectin and VCAM in HUVEC, but not in EA.hy926 (Fig. 4B). This difference between the cell lines was described earlier [41]. SkQ1 suppressed both basic and TNF-induced mRNA expression of ICAM1 (Fig. 3A, 4A), E-selectin, and VCAM (Fig. 4B, C). The effect of SkQ1 was dose-dependent, and its most effective concentration appeared to be 0.2 nM. The classical antioxidants N-acetylcysteine (NAC) (5 mM) and Trolox (0.1 mM) also decreased both basic and TNF-induced mRNA expression level of ICAM1 (Fig. 3B), corresponding to previously published data [42-45]. It is noteworthy that the SkQ1 analogs SkQR1 and SkQBerb, carrying rhodamine-19 and berberine cationic groups, correspondingly [46], had the same activities (data not shown).

### SkQ1 inhibits TNF-induced exposure of ICAM1 at the endothelial cell surface

Adhesion molecules should be exposed at the surface of endothelial cells to fulfill their physiological functions. Cell surface exposition of ICAM1 depends on its synthesis, recycling, and cleavage [47-49]. Cell surface ELISA was applied to analyze the expression of ICAM1 at the surface of EA.hy926 cells after TNF (5 ng/ml) treatment. The highest ICAM1 expression was found 8 hours after the TNF treatment (Fig. 3C). SkQ1 (0.2 nM) and NAC (5 mM) decreased TNF-induced ICAM1 exposure at the cell surface (Fig. 3C).

### SkQ1 suppresses TNF-induced secretion of IL-6 and IL-8 in endothelial cells

TNF activates expression in endothelial cells of proinflammatory cytokines (TNF, IL-6), thus amplifying its own effect, and chemokines (MCP1, IL-8) attracting leukocytes to inflammatory sites. TNF (5 ng/ml) stimulated IL-6 and IL-8 secretion in EA.hy926 cells, while SkQ1 (0.2 nM) and NAC (5 mM) inhibited cytokine secretion (Fig. 3C).

### SkQ1 decreases adhesion of neutrophils to TNF-activated endothelium cells

The main function of ICAM1 is the adhesion of leukocytes to the endothelial surface and promotion of their transmigration into tissues [50]. We studied the process of leukocyte adhesion using endothelial cell line EA.hy926 and neutrophils progenitor cell line HL-60 labeled with BCECF-AM. TNF stimulation of the endothelial cells (50 ng/ml, 18 hours) led to increased adhesion of HL-60 to the endothelial cells (Fig. 3D). The adhesion of HL-60 cells was significantly inhibited by pretreatment with SkQ1 (Fig. 3D).

Thus, we confirmed that *in vitro* SkQ1 suppresses the following TNF-induced proinflammatory features: (i) ICAM1, VCAM, and E-selectin expression; (ii) IL-6 and IL-8 secretion; and (iii) resulting neutrophil adherence. The higher efficiency of mitochondria-targeted antioxidants in comparison with non-targeted ones indicates the important role of mtROS in TNF-induced activation of endothelial cells.

### Mechanisms of antiinflammatory action of SkQ1

**Figure 5:**
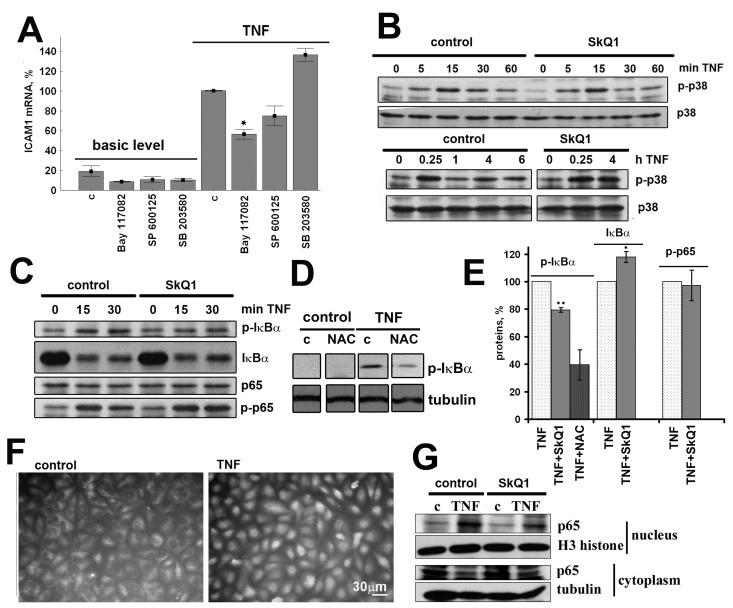
SkQ1 (0.2 nM) inhibits TNF-induced (50 pg/ml) NF-κB activation in EA.hy926 cells; c, control (**A**) Effect of inhibitors of NF-κB (50 mkM Bay 117082), p38 (5 mkM SP 600125) and JNK (20 mkM SB 203580) on TNF-induced ICAM1 mRNA expression. (**B**) Effect of SkQ1 on TNF-induced p38 phosphorylation. (**C**) Effect of SkQ1 on TNF-induced IκBα phosphorylation and proteolysis and p65 phosphorylation. (**D**) Effect of N-acetylcysteine (1 mM) on TNF-induced IκBα phosphorylation. (**E**) Densitometric analysis of protein bands in Figs. 5C and 5D. (**F**) TNF-induced p65 translocation into the nucleus. (**G**) Effect of SkQ1 on p65 content in nuclear and cytoplasmic fractions. Data are represented as mean +/− SEM; n = 3 except for SkQ1 data on Fig. 5E, where n = 5. * p ≤ 0.05, *** p < 0.0001.

NF-κB is the major regulator of TNF-mediated ICAM1, IL-6, and IL-8 expression [7, 8]. Transcription factor AP-1 controlled by stress-activated protein kinases p38 and JNK also contributes to the expression of these proinflammatory molecules [7, 8]. To measure the input ratio of these signaling pathways we used synthetic inhibitors of NF-κB, p38, and JNK (Bay1182, SB203580, and SP600125, respectively). Inhibition of NF-κB resulted in significant (~ 50%) suppression of the TNF-induced ICAM1 expression (Fig. 5A) and completely prevented IL-6 and IL-8 secretion (data not shown). In contrast, inhibition of p38 resulted in ~ 25% decreased expression and secretion of ICAM1 (Fig. 5A). The JNK inhibitor suppressed neither ICAM1 expression nor secretion of IL-6 and IL-8 (Fig. 5A and data not shown).

### SkQ1 does not influence p38 phosphorylation

Activation of p38 depends on its phosphorylation, and TNF triggered rapid transient (5-15 min) followed by subsequent slow (3-4 h) increase in p38 phosphorylation in EA.hy926 cells (Fig. 5B). SkQ1 had no effect on TNF-activated p38 phosphorylation both at 15 minutes and 4 hours (Fig. 5B).

### SkQ1 inhibits NF-κB activation

TNF induces “canonical” NF-κB pathway signaling by recruitment of IκB kinase (IKK) to the TRADD/RIP/TRAF2 signaling complex followed by their phosphorylation and activation in endothelial cells [51]. Activated IKKβ phosphorylates Ser32 of the inhibitory subunit IκBα leading to its proteasomal degradation and subsequent translocation of the released transcriptionally active p65 subunit into the nucleus. In EA.hy926 cells, phosphorylation of IκBα occurred in 5-15 minutes, and its degradation happened in 15-30 minutes, while relocation of p65 into the nucleus was observed 30-60 min after addition of TNF (Fig. 5C, E). SkQ1 pretreatment led to an obvious reduction in p65 content in the cell nuclei (Fig. 5F). The phosphorylation of p65 affects its transcriptional activity [52], and TNF-induced phosphorylation of p65 (Ser536) in EA.hy926 cells was detected in 15 minutes (Fig. 5C). SkQ1 (0.2 nM) inhibited TNF-induced phosphorylation and cleavage of IκBα, as well as the relocation of p65 into the nucleus, but it had no effect on the p65 phosphorylation (Fig. 5C, D, F). The classical antioxidant NAC also inhibited phosphorylation of IκBα according to previously reported data (Fig. 5C, D) [53].

Thus, the antiinflammatory action of SkQ1, at least partially, may be explained by its ability to inhibit the NF-κB activation.

## DISCUSSION

The “inflammaging theory” postulates that aging phenotype can be explained by an imbalance between inflammatory and antiinflammatory networks, which results in low-grade chronic proinflammatory status [54]. The inflammatory vascular reactions are mediated by complex interactions between circulating leukocytes and the endothelium. Proinflammatory cytokines including TNF increase expression of CAMs and leukocyte adhesion followed by invasion through the vascular endothelium. We have shown (Fig. 1, 2) that old mice have increased levels of the vascular inflammatory markers in plasma (TNF and IL-6) and in aorta tissues (ICAM1, VCAM, TNF, and MCP1). Age-related vascular inflammation has been described earlier in mice as well as in humans [36-38]. A significant body of evidence indicates that mitochondrial dysfunction and excessive mtROS production are involved in vascular inflammation and age-related CVDs [55, 56]. Long-term administration of the mitochondria-targeted antioxidant SkQ1 to old mice completely prevented the age-related increase in aortic ICAM1 mRNA expression and attenuated the increase in expression of the other proinflammatory genes (Fig. 1). However, SkQ1 did not affect circulatory TNF and IL-6 levels, thus indicating that mtROS are critical for inflammatory signaling downstream from cytokine expression. Increased expression of CAMs is implicated in early steps of atherosclerosis [57, 58]. The suppression of leukocyte adhesion to endothelial cells by reducing CAM expression prevented development of atherosclerosis [59, 60] and had positive effects on many aseptic inflammatory pathologies [61]. According to our data, mtROS scavenging may attenuate age-related increase in CAM expression and related endothelial dysfunction.

In support of this conclusion, our studies on human endothelial cells *in vitro* demonstrated that mtROS scavenging with SkQ1 downregulated TNF-induced expression of CAMs (Fig. 3A, B, and Fig. 4) and IL-6 and IL-8 secretion (Fig. 3C) and resulting neutrophil adherence to the endothelial cells (Fig. 3D). NAC revealed similar activities, though its efficient concentration was more than seven orders of magnitude higher compared to the mtROS scavenger, this suggesting the important role of mtROS in TNF-induced activation of endothelial cells.

Binding of TNF to the extracellular domain of TNF-R1 leads to activation of TRADD and recruitment of additional adaptor proteins FADD, RIP, and TRAF2 [62]. The latter two are responsible for inflammatory signaling via activation of MAP kinases p38, JNK, and ERK1/2 and transcriptional factor NF-κB [63, 64]. TNF-induced activation of NF-κB relies on phosphorylation and subsequent ubiquitination and degradation of the inhibitory subunit IκB, which retains NF-κB in the cytoplasm of unstimulated cells [65]. The expression of CAMs, IL-6, and IL-8 is substantially controlled by NF-κB [7, 66]. To investigate the molecular mechanism(s) of mtROS-mediated inflammatory response in endothelial cells, we applied specific inhibitors to NF-κB, p38, and JNK. In line with the previously published data, TNF-induced expression of CAMs and IL-6 and IL-8 synthesis depended mainly on NF-κB activity and, to a lesser extent, on p38 (Fig. 5A) [7, 8]. SkQ1 did not affect p38 phosphorylation after TNF stimulation (Fig. 5B) but significantly reduced TNF-dependent translocation of trans-criptionally active NF-κB subunit p65 into the nucleus (Fig. 5F). TNF treatment also led to fast phosphorylation of p65 at Ser536, which could be involved in its activation [52], but this process was not affected by SkQ1 (Fig. 5C, D). The critical event in the NF-κB activation pathway is the phosphorylation of IκBα by the IKK2 complex, which targets it for degradation by the 26S proteasome [67]. SkQ1 inhibited TNF-induced IκBα phosphorylation and degradation (Fig. 5C, D). NAC also inhibited these processes (Fig. 5C, D) according to previously published data [53]. Thus, we have shown that mtROS do participate in TNF-induced inflammatory signal production by inhibiting IκBα phosphorylation and degradation.

Our findings are also consistent with the results obtained by other groups. Alpha-tocopherol and BAY 11-7082 reduced expression of mRNA of CAMs in human aortic endothelial cells activated by TNF [68]. Resveratrol was reported to block the phosphorylation of the p65 subunit of NF-κB, inhibiting its nuclear translocation; however, whether the antioxidant action of resveratrol contributed to its inhibitory effect remains unclear [69]. Administration of another mitochondria-targeted antioxidant, MitoQ, attenuated ischemia–reperfusion-induced increased adhesion molecule expression and enhanced delayed neutrophil infiltration in the liver [70].

It is well known that NF-κB transcription factor is redox-sensitive [71-73]. NF-κB was one of the first transcription factors found to respond upon oxidative stress (H_2_O_2_ or ionizing radiation) [74]. Activators of NF-κB such as TNF or IL-1 led to enhanced ROS production, which contributed to NF-κB activation [75-79]. Compounds with antioxidant properties were shown to block NF-κB activation. However, conflicting data on the role of ROS in NF-κB signaling obtained later in different laboratories confused the situation. It was reported that ROS inhibited NF-κB activation by interfering with its ability to bind DNA [73]. Many effects of presumed antioxidants turned out to be unrelated with their antioxidant potential. TNF-induced NF-κB activation was inhibited by both PDTC and the “classical” antioxidant NAC independently of their antioxidative action: PDTC inhibited IκB ubiquitin ligase, while NAC decreased the affinity of the receptor for TNF [80]. It should be noted, however, that in that study NAC was used at the extremely high concentration of 30 mM. Similarly, modulation of SOD activity also resulted in ambiguous conclusions [81, 82]. The ability of H_2_O_2_ to regulate IKK activity has been investigated by several groups, and opposite results were obtained depending on the cell type [72].

One possible explanation for this controversy is that ROS level can vary significantly among different studies and in some cases exceed a threshold value, thus switching intracellular signaling. Another explanation of this puzzle may be referred to the specific intracellular site of ROS production, which might be critical for the type of cellular response [83]. Though mitochondria are not listed among major sources of ROS in endothelial cells, the importance of mito-chondrial components in inflammatory signaling is now emerging [12]. Our work does not pretend to explain the conflicting data about oxidants and activation of NF-κB; however, it points to an important role of the mitochondria-generated ROS in TNF-dependent activation of NF-κB and the inflammatory response in endothelial cells. Mitochondria-targeted antioxidants are exclusively accumulated in mitochondria and are active at extremely low concentrations, thus greatly reducing the possibility of direct NF-κB inhibition.

In recent years, a large body of evidence has been accumulated indicating mTOR as a key pathway modulating aging and age-related diseases [84]. Excessive activation of mTOR was associated with inflammation and mTOR inhibition by rapamycin was shown to have anti-inflammatory effects in vascular inflammation after angioplasty [85] and atherosclerotic plaques [86]. ROS may function as a potential messenger in mTOR pathway forming positive feedback loop [84]. Interestingly, NAC indirectly inhibited the mTOR pathway [87]. Noteworthy, activation of the pro-survival kinase AMPK also leads to the mTOR inhibition and improves endothelial function [88]. However, it remains unclear whether this pathway is involved in the mtROS-dependent endothelial activation.

Oxidative stress and other stress factors are known to induce anti-inflammatory senescent phenotype in endothelial cells accompanied by reduced expression of the cell adhesion molecules [89]. The cell cycle arrest and anti-inflammatory phenotype was mediated by p38 MAPK [89, 90]. However, SkQ1 at nanomolar concentrations did not induce any morphological signs typical for the senescent cells. Also, SkQ1 neither influenced proliferation of the cells (data not shown), nor induced p38 MAPK phosphorylation (Fig. 5B), thus senescence-related mechanism of its anti-inflammatory action seem improbable.

Numerous studies over the past two decades have clearly implicated an important role for elevated levels of ROS in CVDs. However, analysis of large-scale clinical trials demonstrated that even long-term antioxidant supplement did not reduce mortality from cardiovascular diseases [91, 92]. Therefore, attempts were made to find other targets to combat endothelial dysfunction. One of those promising targets appeared to be endothelial mitochondria [93]. Recently developed mitochondria-targeted antioxidants can be used as promising drug candidates to treat endothelial dysfunction. SkQ1 was shown to possess antioxidant properties in various animal models of diseases associated with oxidative stress [24]. SkQ1 and another plastoquinone-based mitochondria-targeted antioxidant, SkQR1, were also efficient in preventing TNF-induced endothelial cell apoptosis [94]. Our results support the role of mitochondria-derived ROS in NF-κB-mediated inflammatory response both *in vitro* and *in vivo* and open new perspectives in using mitochondria-targeted antioxidants in the prevention or treatment of age-related CVDs.

In conclusion, this study demonstrates that TNF-induced mtROS participates in activation of CAMs mediated by NF-κB in endothelial cells and suggests that vascular aging can be, at least partially, prevented by mitochondria-targeted antioxidants.

## METHODS

### Animals and SkQ1 treatment

The experimental group (n = 10) contained 24-month-old F1 (CBAxC57Bl/6) mice after long-term (8 months) administration of antioxidant SkQ1 (100 nmol/kg body weight per day) with drinking water. The control groups included 24-month-old (n = 12) and 6-month-old (n = 12) F1 (CBAxC57Bl/6) mice without SkQ1 treatment. All of the animals were kept in plastic cages under standard temperature, light, and feeding regimes. At the end of the experiment, the mice were sacrificed. All animal care and experimental procedures were in compliance with European Directive-2010 of FELASA.

### Cell cultures

The human endothelial cell line EA.hy926 cells were cultured in DMEM (Dulbecco's modified Eagle's medium) (Gibco, USA) supplemented with 10% fetal bovine serum (FBS) (HyClone, USA) and with HAT (Sigma). Human umbilical vein endothelial cells (HUVECs) kindly provided by M. A. Lagarkova (Vavilov Institute of General Genetics, Moscow) were grown in EGM-2 BulletKit (Lonza) and used at passages 2-4. Flasks and 6-well plates used for HUVEC culturing were pretreated with 2% gelatin. Human promyelocytic leukemia cell line (HL-60) cells were grown in RPMI 1640 medium supplemented with 10% fetal bovine serum (FBS) (HyClone, USA). All cell cultures were incubated at 37°C in 5% CO_2_.

### ELISA

Mouse blood plasma and growth medium cytokine IL-6, IL-8, and TNF contents were determined in cell-free supernatants with commercial ELISA kits (eBioscience) according to the manufacturer's instructions.

### Cell-surface ICAM1 expression

EA.hy926 cells were grown in 96-well cell-culture plates at 10,000 cells per well. After adhesion and spreading, the cells were treated with SkQ1 (0.2 nM). Endothelial cells were incubated with TNF (courtesy of Dr. L. N. Shingarova, Institute of Bioorganic Chemistry, Moscow) (5 ng/ml, 8 hours), fixed with 2% paraformaldehyde, and cell-surface ICAM1 was detected with antibody to human ICAM1 (eBioscience) and secondary antibody conjugated to HRP (Sigma). The peroxidase reaction was carried out in buffer (0.05 Trisodium citrate, 0.1M Na_2_HPO_4_**·**12H_2_O, pH = 5.0) with 0.03% hydrogen peroxide and 0.5 mg/ml ortho-phenylenediamine. The reaction was blocked with 10% H_2_SO_4_. Absorbance was measured using a Multiskan EX Microplate Photometer (Thermo Scientific) at 495 nm.

### HL-60 cells adhesion

EA.hy926 cells were cultured in 24-well cell-culture plates at 100,000 cells per well. Endothelial cells were incubated with TNF (5 ng/ml, 8 hours). The culture medium was removed, and the endothelial cell monolayer was washed twice with warm RPMI 1640. BCECF-labeled HL-60 suspension was added to the endothelium, and the cells were incubated for 30 min at 37°C in 5% CO_2_. Non-adherent cells were removed by washing five times with warm RPMI 1640. Then the cells were fixed with 2% paraformaldehyde. HL-60 adhesion was measured using microscopic analysis on an Axiovert microscope equipped with 20x objective (Carl Zeiss, Jena, Germany). Microscopy images were processed using the public domain ImageJ software (National Institutes of Health, http://imagej.nih.gov/)

### Western blot analysis

EA.hy926 cells were cultured in 6-well cell-culture plates at 200,000 cells per well. The cells were lysed in buffer (62.5 mM Tris-HCl, pH 6.8, 2% SDS, 10% glycerol, 50 mM DTT, 0.01% bromophenol blue). Equal amounts of protein were separated onto 12% SDS polyacrylamide gels and then transferred to PVDF membranes (Amersham, USA). Membranes were probed with antibodies against p-38, p-p38, IkBα, p-IkBα, p65, p-p65, H3 histone (Cell Signaling), and tubulin (Sigma Aldrich, USA). The blots were developed with appropriate secondary antibody conjugated to HRP (Sigma Aldrich, USA). The membranes were treated with HRP-conjugated secondary antibody (Sigma) and developed with ECL chemiluminescence reagents (Amersham) according to the manufacturer's protocol. The ImageJ software was used for densitometric analysis of the bands.

### Immunofluorescence microscopy

EA.hy926 cells were grown on glass coverslips placed in 6-well cell-culture plates at 200,000 cells per well. Confluent monolayers of endothelial cells were incubated with TNF (0.5 ng/ml, 30 min), fixed using 2% paraformaldehyde, and treated with 1% Triton X-100. Fixed cells were incubated with antibody to p65. TRITC-conjugated antibodies against rabbit immunoglobulins (Jackson Labs, Bar Harbor, ME) were used as secondary antibodies. Images were acquired using an Axiovert microscope equipped with 40x objective (oil immersion Neofluar) (Carl Zeiss, Jena, Germany).

### Nuclear extracts

EA.hy926 cells were collected, washed with cold PBS, spun down for 5 min at 500*g* at 4°C, and resuspended in Sucrose Buffer (0.32 M Sucrose, 10 mM Tris HCl pH 8.0, 3 mM CaCl_2_, 2 mM MgOAc, 0.1 mM EDTA, 0.5% NP-40, 1 mM DTT, and 0.5 mM PMSF). The cytoplasmic fraction was separated, and the nuclear pellet was washed with 1 ml of Sucrose Buffer without NP-40. The pellets were resuspended in Low Salt Buffer (20 mM HEPES (pH 7.9), 1.5 mM MgCl_2_, 20 mM KCl, 0.2 mM EDTA, 0.5 mM DTT, and 0.5 mM PMSF). An equal volume of High Salt Buffer (20 mM HEPES (pH 7.9), 1.5 mM MgCl_2_, 800 mM KCl, 0.2 mM EDTA, 1% NP-40, 0.5 mM DTT, 0.5 mM PMSF) was added. The samples were incubated for 30-45 min at 4°C on a rotator, and debris was spun down at 14,000*g* for 15 min at 4°C.

### Quantitative real-time PCR (qRT-PCR)

Total RNA was isolated from cultured cells and mouse aortas using a Qiagen RNeasy Mini Kit (Qiagen, Inc. Valencia, CA) according to manufacturer's protocol. The RNA quality was assessed by measuring the A260/A280 nm absorption ratio. After DNase treatment (Fermentas), cDNA was obtained by annealing 2 μg of denatured total RNA with 0.1 μg of random hexamers and 0.1 μg of Oligo-dT. The mixture was then incubated with 200 units of Superscript III reverse transcriptase (Invitrogen) for 50 min at 43°C. The qRT-PCR was carried out using an iCycler iQ real-time PCR detection system (Bio-Rad, Hercules, CA, USA). For detection of target genes, EVA Green master mix (Syntol, Russia) was used according to the manufacturer's instructions. Primer sequences are listed in the Supplementary Table 1. The thermal profile for EVA Green qRT-PCR included an initial heat-denaturing step at 95°C for 3 min and 45 cycles at 95°C for 15 s, an annealing step (Supplementary Table 1) for 30 sec, and 72°C for 30 sec, coupled with the measurement of fluorescence. Following amplification, the melting curves of PCR products were monitored from 55 to 95°C to determine the specificity of amplification. Each sample was run in triplicate, and a non-template control was added to each run. PCR efficiency (E) was calculated according to the equation E = 10(^−1^/slope) using standard curves. Target-gene mRNA levels were corrected for corresponding reference gene(s) RPL32 and GAPDH. The data are represented as the mean of at least three independent experiments ± SEM.

### Statistical analysis

The data are expressed as mean ± SEM. Groups were compared with a Student's *t*-test using the Statistica 6.0 software.

## SUPPLEMENTARY DATA


