# Supplementary material for: Role of mitochondrial reactive oxygen species in age-related inflammatory activation of endothelium
Source: Aging (Albany NY). 2014 Aug 13;6(8):661–74. doi: 10.18632/aging.100685 (PMC4169860; doi:10.18632/aging.100685)
Supplement: Supplementary file 1 [file aging-06-661-s001.pdf]

SUPPLEMENTARY DATA

Supplementary Table 1. Primer sequences for qRT-PCR.

Target gene	Orga-nism	Forward (5'-3')	Reverse (5'-3')	Annealing temperature, ^oC
ICAM1	*Mouse*	CAGGATATACAAGTTACAGAAGG	TGACAGCCAGAGGAAGTG	56
TNF	*Mouse*	CGTGGAACTGGCAGAAGAG	ACAAGCAGGAATGAGAAGAGG	58
VCAM	*Mouse*	GTGGAAATGTGCCCGAAAC	GAGCCAACTTCAGTCTTAGATTC	54
MCP-1	*Mouse*	CACTCACCTGCTGCTACTCATTC	GCTTCTTTGGGACACCTGCTG	57
RPL32	*Mouse*	GGCACCAGTCAGACCGATATG	CCTTCTCCGCACCCTGTTG	56
GAPDH	*Mouse*	TCCTGGAAGATGGTGATGGGATTT	TGGTCACCAGGGCTGCTTTTA	54
ICAM1	*Human*	TGTCATCATCACTGTGGTAGC	CTTGTGTGTTCGGTTTCATGG	56
VCAM	*Human*	CTTCTCGTGCTCTATTTTG	TTGACTTCTGTGCTTCTAC	54
E-selectin	*Human*	TGGTTGAGTGTGATGCTGTG	CGTTGGCTTCTCGTTGTCC	58
RPL32	*Human*	CATCTCCTTCTCGGCATCA	AACCCTGTTGTCAATGCCTC	56

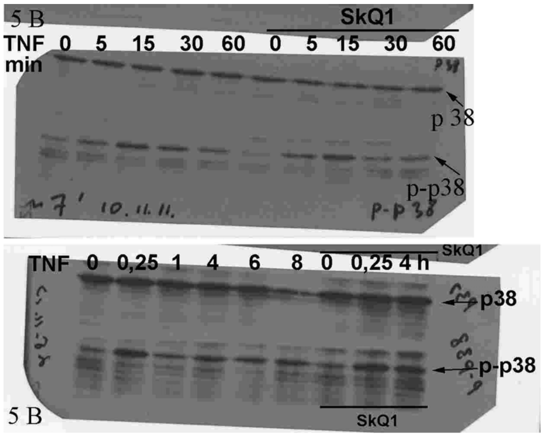

Supplementary Data to Figure 5B

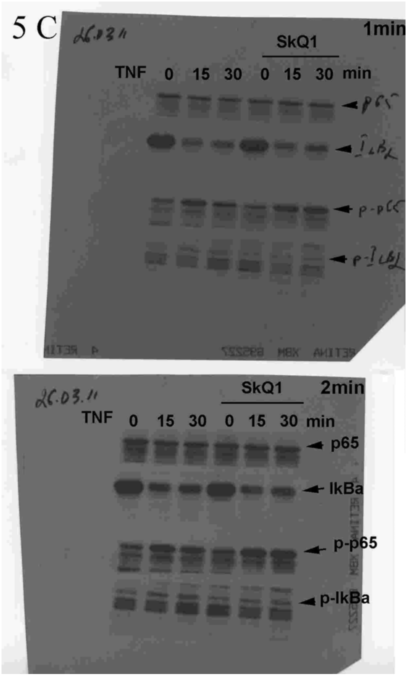

Supplementary Data to Figure 5C

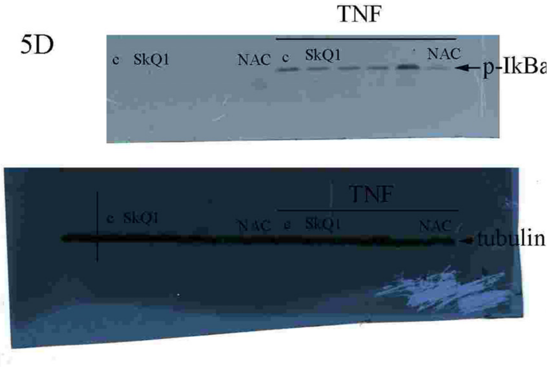

Supplementary Data to Figure 5D

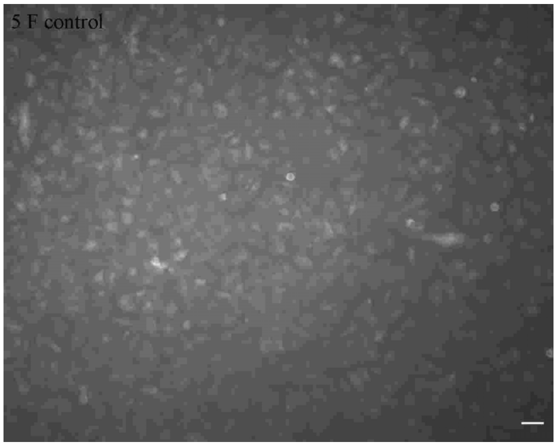

Supplementary Data to Figure 5F

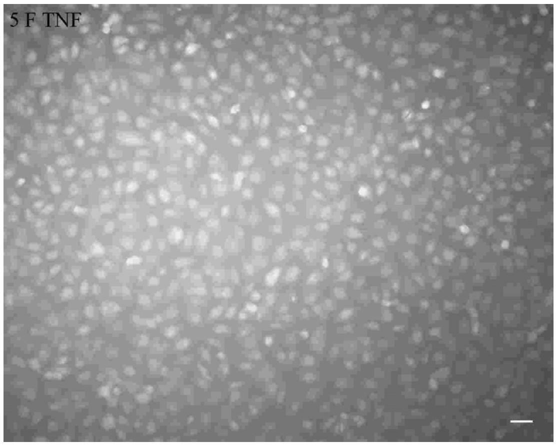

Supplementary Data to Figure 5F

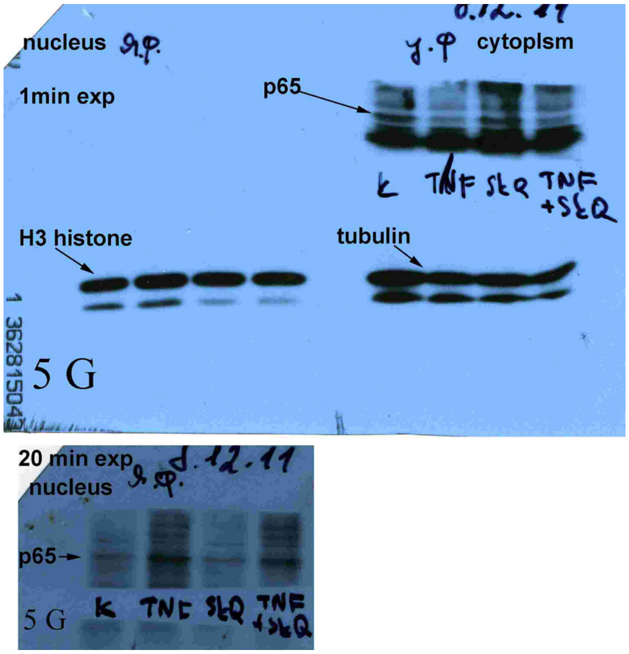

Supplementary Data to Figure 5G
